# Effect of assisted reproductive technology on the molecular karyotype of missed abortion tissues

**DOI:** 10.1042/BSR20180605

**Published:** 2018-10-15

**Authors:** Gang Li, Haixia Jin, Wenbin Niu, Jiawei Xu, Yihong Guo, Yingchun Su, Yingpu Sun

**Affiliations:** Reproductive Medical Center, First Affiliated Hospital of Zhengzhou University, Zhengzhou, China

**Keywords:** assisted reproductive technology (ART), missed abortion, molecular karyotype, Single nucleotide polymorphism (SNP)

## Abstract

Missed abortion is one of the common complications of assisted reproductive technology (ART). Genetic abnormality is the most important factor. However, the effect of ART on the molecular karyotype of products of conception (POC) remains unknown. We explored the effect of ART on the molecular karyotype of POC in miscarriage. POC were obtained from women undergoing ART. Single nucleotide polymorphism (SNP) microarray was used to analyze the molecular karyotype. A total of 1493 POC were collected for SNP array analysis. The total rate of karyotypic abnormalities was 63.1% (943/1493). The proportion of karyotypic abnormalities was 70.4% (193/416) in >35-year-old group, which was significantly higher than that (60.6%) (343/566) in <30-year-old group and that (60%) (307/511) in the 30–35-year-old group. In natural conception (NC) group, the proportion of karyotypic abnormalities was 64.6% (201/311), whereas in ART group it was 62.7% (742/1182) and, there was no significant difference. The ratio between male and female fetuses was 1:1.13 (698/795). The rate of karyotypic abnormalities in male was 62.9% (439/698) and that in female was 63.4% (504/795), and these values did not differ significantly (*P*=0.84). Molecular karyotypic abnormality is the most important reason in miscarriage, and female age is a significant factor influencing the karyotypic abnormalities. Comparison with NC, ART, and gender of aborted embryos may not increase the rate of molecular karyotypic abnormality in miscarriage.

## Introduction

With the development of assisted reproductive technology (ART) and its wide applications, the pregnancy rate has greatly improved, but the live birth rate has not significantly improved, raising increasing attention on the clinical outcomes of ART pregnancy. Missed abortion is one outcome of pregnancy and one of the common complications of ART. Missed abortion not only reduces the clinical pregnancy rate of ART but also increases the psychological and economic burden of couples undergoing ART treatment. Therefore, attention to missed abortion in ART pregnancy, analysis of the possible causes, and application of effective prevention and treatment measures are of great significance in increasing the clinical ART pregnancy rate, reducing birth defects, and improving the clinical prognosis.

The reason for missed abortion is very complex, and the pathogenesis is still not entirely clear. Amongst the many factors, chromosomal abnormality is the most important factor. At present, the methods for evaluating the chromosomes of spontaneously aborted embryos are mainly as follows: karyotype analysis of chorionic villous cells, fluorescence *in situ* hybridization (FISH), and chromosomal microarray analysis (CMA). CMA is an advanced genetic detection method that includes the array comparative genomic hybridization (aCGH) and single nucleotide polymorphism (SNP) microarray techniques. At present, CMA is recommended as the preferred method for the detection of congenital dysplasia and congenital malformations by the International Standards for Cytogenomic Arrays Consortium [1].

In the present paper, we conducted SNP microarray detection and retrospective analysis of 1394 cases of aborted embryonic tissues from missed abortion patients obtained by uterine curettage from the Reproductive Medicine Center of the First Affiliated Hospital of Zhengzhou University from November 2011 to May 2017. The present study was performed to explore the causes of missed abortion and the impact of ART on molecular karyotype in missed abortion, aiming at improving the live birth rate of ART pregnancy and proposing feasibility recommendations.

## Materials and methods

All study methods were approved by Institutional Review Board and Ethics Committee of the First Affiliated Hospital of Zhengzhou University (number 2011-012) and were performed in accordance with the World Medical Association Declaration of Helsinki. All subjects enrolled in the study gave written formal consent to participate.

### Materials

Between November 2011 and May 2017, patients experiencing early pregnancy missed abortion underwent a dilation and curettage (D&C) in our reproductive medical center. The 26 patients with twin abortion were excluded from the study. Products of conception (POC) were collected from 1493 couples of singleton miscarriage for SNP array analysis. All the couple’s cytogenetic karyotypes (G-band) were normal. Of the 1493 cases, 311 experienced natural conception (NC) and 1182 were pregnant by ART. Of the 1182 cases, 93 underwent artificial insemination (AI), 534 fresh *in vitro* fertilization (IVF)-embryo transfer, 151 fresh intracytoplasmic sperm injection (ICSI)-embryo transfer, and 404 thawed embryo transfer. The NC group was used as a strong internal control for data analysis.

Ovarian stimulation of IVF/ICSI was performed with standard long protocol. After complete down-regulation Gonal-F (Follitropin alfa for injection, EMD Serono,Inc.) was given. When the maximal follicle was more than 20 mm and the follicles >16 mm accounted for more than two-thirds of total follicles, human chorionic gonadotropin (hCG) was given. Thirty-seven hours later, oocyte retrieval was performed under ultrasonic guidance. Two to five days later, one to three embryos or one blastocyst was transferred. The outcome of pregnancy was recorded and early missed abortion cases were enrolled.

### Methods

All chorionic villi or fetus tissue were obtained from all the cases. Under anatomic microscope, chorionic villi or fetus tissues were obtained. The chorionic villi or fetus tissues were washed with phosphate buffer solution (PBS) to remove coagulated blood and decidua, and then stored at −80°C for DNA extraction.

### DNA extraction

Sample DNA was extracted with QIAamp DNA Mini Kit (Qiagen, Hilden, Germany) according to the manufacturer’s instructions. The extracted DNA was quantitated with NanoVue Plus (GE, Fairfield, Connection, U.S.A.), and then stored at −20°C.

### SNP microarray analysis

DNA (200 ng) was used as an input for a single array. DNA amplification, tagging, and hybridization were performed according to the manufacturer’s protocols. The arrays were scanned on a HiScanSQ (Illumina, U.S.A.). Data analysis was performed using GenomeStudio (Illumina, standard settings). The HumanCytoSNP-12v.21 array, which covers more than 220000 markers, was employed in the present study to detect molecular karyotype, and the raw data were analyzed using GenomeStudio software (Illumina). Two independent parties analyzed all of the data, and the results were presented under strict criteria.

### Statistical analysis

For analysis, SPSS12.0 software (version 9, SPSS Inc., Chicago, U.S.A.) was used. The results are expressed as the mean ± S.D. The *P*-values less than 0.05 were considered significant. Chi-square test was used to test the frequencies and Student’s *t*test was used for comparing the means.

## Results

From November 2011 to May 2017, a total of 1493 patients who sought treatment for missed abortion were treated with D&C surgery under intravenous anesthesia. The karyotypes of all couples were normal. The aborted villous tissues and embryonic tissues were subjected to SNP microarray analysis. Diagnostic results from the SNP molecular karyotype were obtained for all specimens with a detection success rate of 100 %. The average female age was 32.1 ± 5.3 years, and the average male age was 33.3 ± 6.5 years (Table 1). Amongst the 1493 specimens, molecular karyotypic abnormalities were detected in 943 cases. The detection rate of karyotypic abnormalities was 63.2% (943/1493).

**Table 1.** Basic characteristics of couples in the study

Characteristic	Total	NC + AI	IVF	ICSI
Included couples, number	1493	404	772	317
Female age, years	32.1 (5.3)	31.1 (5.0)	32.9 (5.4)	32.3 (5.2)
BMI, kg/m^2^	21.9 (3.2)	20.9 (3.3)	22.2 (3.2)	22.3 (3.1)
Male age, years	33.3 (6.5)	31.6 (6.3)	34.2 (6.7)	32.8 (6.4)
BMI, kg/m^2^	24.8 (3.8)	24.7 (3.7)	24.8 (3.9)	24.9 (4.1)
Previous miscarriage				
0	44%	25%	51%	76%
1	33%	45%	33%	19%
≥2	23%	30%	11%	5%

Data given as mean (S.D.) unless otherwise indicated.

### Comparison of karyotypic abnormalities in POC of different age groups

There were 293 cases of karyotypic abnormalities in the >35-year-old group. The rate of karyotypic abnormalities was 70.4% (293/416), which was significantly higher than those of the <30-year-old group (60.6% (343/566) and the 30–35-year-old group (60% (307/511), respectively. The difference was found to be statistically significant by means of the chi-squared test (*P*=0.001, *P*=0.001). However, there was no significant difference in the rate of karyotypic abnormalities between the <30-year-old group and the 30–35-year-old group (*P*=0.86) (Table 2).

**Table 2.** Abnormal molecular karyotype in different age groups

Age (years)	*n*	Normal molecular karyotype ( %)	Abnormal molecular karyotype ( %)
<30	566	223 (39.4%)	343 (60.6%)
30-35	511	204 (40%)	307 (60%)
>35	416	123 (29.6%)	293 (70.4%)^1^^,^^2^
Total	1493	550 (36.8%)	943 (63.2%)

1 *P*=0.001 compared with <30-year-old group.

2 *P*=0.001 compared with <30-year-old group.

### Effects of different assisted reproductive techniques on the molecular karyotype of POC tissues

**Figure 1:**
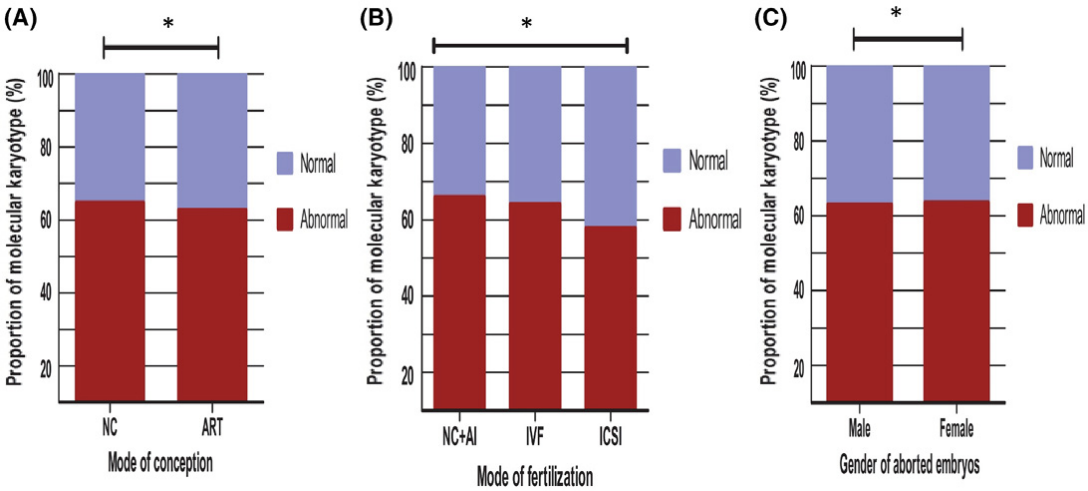
Effect of ART on the molecular karyotype of POC There is no significant difference in the rate of karyotypic abnormalities between the NC and ART groups (**A**). As for abnormal molecular karyotype in different modes of fertilization, there was no statistically significant difference amongst the NC + AI, IVF, and ICSI groups (**B**). The ratio between aborted male and female embryos was approximately 1:1.13. The rate of karyotypic abnormalities in male and female embryos was 62.9 and 63.4%, respectively. The difference between these two values was not statistically significant (**C**). *P* was calculated by Chi-square test analysis; *P>0.05.

In the 311 cases of NC, the proportion of karyotypic abnormalities was 64.6% (201/311) compared with 1182 cases in the ART group, where the rate was 62.7% (742/1182). The difference between the two was not significant (*P*=0.54) (Figure 1).

In addition, we compared the effects of different fertilization methods on the molecular karyotype of miscarriage tissues. In the 404 cases of combined NC (311 cases) and AI (93 cases) groups, the proportion of karyotypic abnormalities was 65.8%. In the 772 cases treated with IVF, the percentage of karyotypic abnormalities was 64%, and in the 317 cases treated with ICSI, the proportion of karyotypic abnormalities was 57.7%. There was no statistically significant difference between the three groups (Figure 1B).

### Sex of spontaneously aborted embryos and karyotypic abnormalities

The ratio between aborted male and female embryos was approximately 1:1.13 (698/795) (Figure 1C). The rate of karyotypic abnormalities in male and female embryos was 62.9 and 63.4%, respectively. The difference between these two values was not statistically significant (Table 3). However, for missed abortion in the first trimester, the proportion of female embryo was higher than male embryo (1.13:1).

**Table 3.** Abnormal molecular karyotype in male/female group

Gender of aborted embryos	*n*	Normal molecular karyotype (%)	Abnormal molecular karyotype (%)
Male	698	259 (37.1%)	439 (62.9%)^1^
Female	795	291 (36.6%)	504 (63.4%)^2^
Total	1493	550 (36.8%)	943 (63.2%)

1 *P*=0.08 compared with female group.

2 Contained 52 cases of 45, X.

## Discussion

### Effect of ART on the molecular karyotype of POC

In the general population, the missed abortion rate for NC is 10–15% [2]. However, whether the proportion of spontaneous abortion for ART pregnancy is higher than that for NC is still controversial. Due to different sample sizes and the differences in age and the definition of spontaneous abortion, the incidence rate of missed abortion in ART pregnancy is not consistent in different reports. According to the U.S. Centers for Disease Control and Prevention (CDC), in 62228 cases of clinical ART pregnancy, the spontaneous abortion rate is 14.7%, which does not differ significantly from the spontaneous abortion rate for NC in the 12–44-year-old population, suggesting that ART treatment does not increase the rate of spontaneous abortion [3]. Additionally, the missed abortion rate for ART pregnancy was reported to be between 18 and 30% by a number of reproductive centers, which is significantly higher than that for NC [4,5]. The correlation coefficient between ART pregnancy and the risk of spontaneous abortion was 1.20 (95% confidence interval (CI): 1.03–1.46) [6]. There is a difference between the missed abortion rates for ART pregnancy and NC probably because missed abortion in ART pregnancies are detected and reported, whereas missed abortion in NC may be difficult to monitor or may be underestimated.

**Figure 2:**
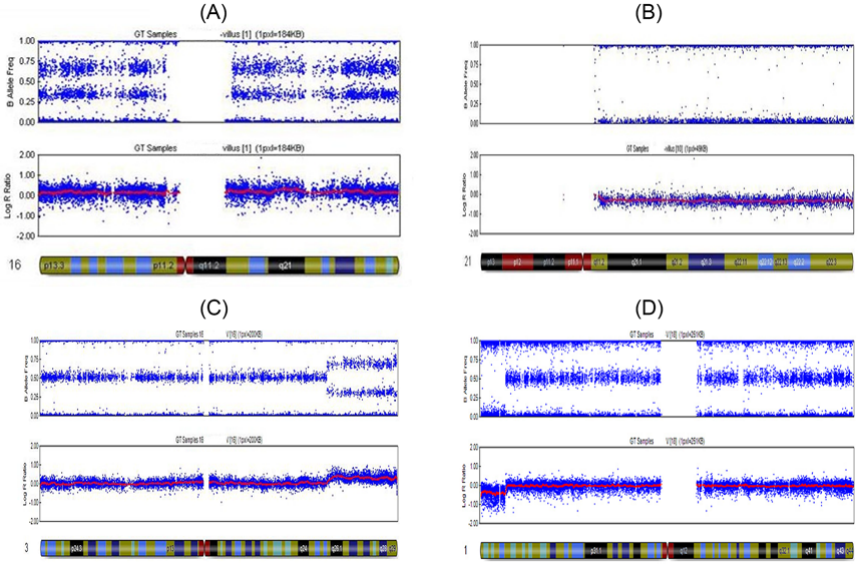
Molecular karyotype of POC using SNP microarrays (**A**) Displays the trisomy chromosome 16 diagnostic reading obtained from miscarriage, AAA, AAB, ABB, and BBB alleles, and a significant shift in the smooth log R ratio is observed, consistent with the trisomy karyotype. (**B**) Shows the monosomy chromosome 21 diagnostic reading obtained from POC, AA, and BB alleles observed without AB and a significant shift in the smooth log R ratio is observed, consistent with the monosomy karyotype. (**C**) Demonstrates the duplication of q25.32→q29 reading of chromosome 3. AAA, AAB, ABB, and BBB alleles are observed from q25.32 to q29. A significant shift in the smooth log R ratio is observed from q25.32 to q29 of chromosome 3. (**D**) Presents the deletion of p26.1→pter reading of chromosome 1. AA, AB, and BB alleles are observed from p26.1 to qter of chromosome 1, however, AA and BB alleles are observed without AB from p26.1 to pter of chromosome 1 represented. A significant shift in the smooth log R ratio is observed from p26.1 to pter of chromosome 1.

Molecular karyotypic abnormality is an important factor in missed abortion, and its influencing factors are also numerous, especially in ART. Our preliminary small-sample (*n*=81) retrospective study suggested no significant differences between the NC and ART groups in terms of molecular karyotypic abnormalities in POC tissues [7]. In this study, we increased the sample size (*n*=1493) and still obtained the same results. This finding suggests that ART does not increase the rate of karyotypic abnormalities in the aborted tissues of missed abortion. This conclusion is consistent with the reports in the literature [8–10]. For different fertilization methods, a study showed that the rate of chromosomal abnormalities increased in the POC tissues of ICSI pregnancy [11]. Another study showed that the proportions of chromosomal abnormalities in the POC tissues of ICSI and IVF pregnancy abortion were comparable, but the proportion of chromosomal abnormalities was higher in the POC tissues of ICSI pregnancy [12]. Our early small-sample (*n*=81) study showed that the ICSI group had a significantly lower rate of karyotypic abnormalities than the NC + AI and IVF groups. However, in the present study with a larger sample size, the rates of karyotypic abnormalities in the NC + AI, IVF, and ICSI groups were 65.8, 64, and 57.7%, respectively, suggesting no statistically significant difference amongst the three groups. This result indicates that the ICSI procedure does not increase the rate of chromosomal karyotypic abnormalities in the POC tissues of missed abortion. However, in the three groups, the ICSI group had the lowest rate of karyotypic abnormalities, which indicated that the cause of missed abortion in the ICSI group was more complicated than those in the IVF and NC + AI groups. Our data showed that the most common type of karyotypic abnormalities was autosomal trisomy, followed by complex abnormalities, triploidy, monosomy X etc.(Table 4 and Figure 2). This conclusion requires larger sample studies to confirm.

**Table 4.** Type of abnormal molecular karyotype in abortion tissue

Molecular karyotype	ART (%)	NC (%)	Total (%)
Autosomal trisomy	487 (65.6%)	110 (54.7%)	597 (63.3%)
Deletion	19 (2.6%)	4 (2.0%)	23 (2.4%)
Duplication	37 (4.9%)	12 (5.9%)	49 (5.2%)
Triploidy	42 (5.6%)	21 (10.4%)	63 (6.7%)
Autosomal monosomy	18 (2.4%)	0 (0)	18 (1.9%)
Monosomy X	35 (4.7%)	17 (8.5%)	52 (5.5%)
Mosaic	27 (3.6%)	8 (4.0%)	35 (3.7%)
Uniparental disomy (UPD)	16 (2.2%)	12 (5.9%)	28 (3.0%)
Complex abnormalities	61 (8.2%)	17 (8.5%)	78 (8.3%)
Total	742	201	943

### Factors related to missed abortion

Spontaneous abortion is a common complication of pregnancy, and missed miscarriage is a special type of spontaneous abortion. Most abortions that occur during the gestational age of 9–12 weeks are missed abortion, and the fetus has been dead in the uterus for several weeks. However, due to the blood HCG test and B-ultrasound examination following embryo transfer, the occurrence of a missed abortion can be detected earlier in ART pregnancy.

The causes of spontaneous abortion are complex, and the underlying causes include genetics, anatomy, immunization, endocrine factors, infection, thrombotic tendency, and even the environment and other unknown factors. Moreover, as for ART, the reason is more complex. Underlying etiology of infertility and treatment can contribute to the risk of spontaneous abortion. For example, the PCOS patients have higher miscarriage rates than the normal women. Other factors may include the protocol of controlled ovarian stimulation, embryo micromanipulation, multiple pregnancy etc. However, the underlying etiology of infertility is complicated, and it is difficult to confirm the clear underlying reason. Genetic factors account for 50–60% of all factors relevant to spontaneous abortion. Particularly for early abortion (gestational age <3 months), more than 50% of embryos have chromosomal karyotypic abnormalities, and the causes of 37–79% of clinical spontaneous abortions remain unclear. Therefore, it is necessary to perform genetic analysis of POC to find the probable cause, despite the fact that POC genetic analysis is mostly placental rather than fetal tissue and it may introduce diagnostic inaccuracy. Clinically, the most commonly identified cause of missed abortion is chromosomal abnormality. The traditional detection methods for chromosomal abnormality include cell culture using POC and G-banding analysis. Due to the limitations of traditional methods, new methods, such as aCGH and SNP array, were developed, and these techniques were better at detecting the molecular karyotype of tissues. Chromosome microarray analysis (such as CGH and SNP) is a first-line method for the clinical detection of individual developmental disorders and congenital dysplasia and for prenatal diagnosis. In the present paper, SNP microarray was used to analyze the molecular karyotype of POC; the diagnostic rate was 100%, and the rate of karyotypic abnormalities was 63.2% (943/1493). SNP microarray still had a great advantage in analyzing the molecular karyotype of POC [13].

Female age is an important factor influencing the karyotypic abnormalities in POC. The risk of aneuploidy pregnancy increases with increases in female age. An advanced maternal age is the only factor that has been identified to be closely related to the risk of embryonic chromosomal abnormalities. The results of the present study also showed that the rate of karyotypic abnormalities was 70.4% in the POC of the >35-year-old group, which is significantly higher than that of the <30-year-old group (60.6%) and that of the 30–35-year-old group (60%). Therefore, for women of advanced age (>35 years old), prenatal diagnosis is very necessary. For elderly infertile couples, preimplantation genetic screening (PGS) may be a good choice because PGS can select embryos with normal molecular karyotypes for transfer. With the development of high-throughput whole-genome chromosome testing techniques, such as microarray and NGS, and the improvements in vitrification technology, PGS2.0, characterized by TE cell biopsy and high-throughput detection of all chromosomes, is considered to improve clinical pregnancy rates, reduce abortions, and improve clinical pregnancy outcomes, although the debate is still going on. SNP microarray technology has been used in this country and abroad for PGS on elderly couples; the results show that PGS can reduce the risk of miscarriage after pregnancy and improve the prognosis of ART pregnancy [14,15].

The present study included 698 cases of male embryos and 795 cases of female embryos, with a male/female ratio of 1:1.13. There were 943 embryos with karyotypic abnormalities, with male and female embryos accounting for 439 and 504 cases, respectively; the rate of male and female embryos with karyotypic abnormalities was 62.9 and 63.4%, respectively. Although the difference in the proportion of male and female embryos with karyotypic abnormalities was not statistically significant (*P*=0.84), the proportion of female embryos was higher than the proportion of male embryos for spontaneous abortion, and this result is consistent with other studies reporting that risk of spontaneous abortion is higher for female embryos than for male embryos in early pregnancy. Yusuf and Naeem [16] believed that in the early stages of pregnancy, the possibility of abortion is higher in female embryos than in male embryos and that with increases in the gestation weeks, the possibility of missed abortion decreases in female embryos but increases in male ones. There may be a mechanism causing female embryos to become prone to spontaneous abortion in early pregnancy, but this gender-specific mechanism remains unclear and may be related to X-chromosome inactivation [17–19] and X-linked recessive lethal mutations [20,21].

In summary, the causes of missed abortion are complex in NC and ART pregnancy [22]. Whether ART affects the molecular karyotype of POC tissues is also an important aspect of ART safety. The present paper shows that embryonic karyotypic abnormality is one of the most important causes of missed abortion and that female age is an important factor affecting embryonic karyotypic abnormalities. Compared with NC, the ART itself and the sex of the embryo did not increase the rate of embryonic karyotypic abnormalities in POC tissues. The samples of NC in the present study came from a subpopulation of patients attending the Reproductive Medical Centre. They are not entirely enrolled from general population. However, to the best of our knowledge, our study has the largest number of ART samples amongst studies that have analyzed the molecular karyotype of missed abortion tissues using SNP microarray technology; however, the number of samples is still limited, and more samples are needed in further studies to confirm our conclusion.

## Abbreviations

aCGH: array comparative genomic hybridization
AI: artificial insemination
ART: assisted reproductive technology
CMA: chromosomal microarray analysis
hCG/HCG: human chorionic gonadotropin
ICSI: intracytoplasmic sperm injection
IVF: *in vitro* fertilization
NC: natural conception
NGS: next generation sequencing
PGS: preimplantation genetic screening
PCOS: polycystic ovarian syndrome
POC: product of conception
SNP: single nucleotide polymorphism
TE: trophectoderm
